# The Association of VDR *FokI* T>C (rs2228570) Gene Variants With T2DM, and Its Complications: Influence on BMI, Oxidative Stress, and Dyslipidemia

**DOI:** 10.1002/jcla.70090

**Published:** 2025-09-29

**Authors:** Armin Sharifi, Mahsa Nouri, Ebrahim Shakiba, Zahra Ghorbani, Zohreh Rahimi

**Affiliations:** ^1^ Department of Clinical Biochemistry, Medical School Kermanshah University of Medical Sciences Kermanshah Iran; ^2^ Student Research Committee, Medical School Kermanshah University of Medical Sciences Kermanshah Iran; ^3^ Medical Biology Research Center, Health Technology Institute Kermanshah University of Medical Sciences Kermanshah Iran

**Keywords:** BMI, diabetes complications, *FokI* gene variants, oxidative stress, T2DM

## Abstract

**Introduction:**

The present study aimed to investigate the association between vitamin D receptor (VDR) *FokI* gene variants and the risk of type 2 diabetes mellitus (T2DM) and its complications, as well as with oxidative stress parameters.

**Methods:**

We investigated 300 individuals with diabetes with and without neuropathy and retinopathy, and 100 individuals without diabetes. The PCR‐RFLP technique was used to determine the genotypes of VDR *FokI* T>C (rs2228570). The oxidative stress parameters were measured using chemical methods.

**Results:**

In individuals with diabetes, there were significantly higher levels of body mass index (BMI) and oxidative stress than in controls. The presence of the FokI CC genotype and the C allele were associated with 2.46 times and 1.6‐fold increased risk of T2DM, respectively, and enhanced diabetic neuropathy risk by 3.41‐ and 3.68‐fold, respectively, and elevated diabetic retinopathy risk by 2.45 and 1.59 times, respectively. The presence of FokI TC + CC compared to the TT genotype resulted in higher triglycerides, total oxidative status, and BMI levels in individuals with diabetes. We found significantly higher BMI, oxidative stress index, and significantly lower levels of total antioxidant capacity in females than in males among individuals with diabetes and controls.

**Conclusion:**

This study indicates that the *FokI* CC genotype and the *FokI* C allele are associated with an increased risk of T2DM and its complications. We observed the influence of VDR *FokI* variants on dyslipidemia, oxidative stress, and BMI. It seems the risk factors of developing T2DM, obesity, and oxidative stress among women are more prevalent than in men.

## Graphical abstract

*FokI* gene variants, oxidative stress and the risk of type 2 diabetes mellitus.

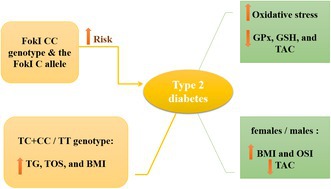

## 1 Introduction

Type 2 diabetes mellitus (T2DM) is one of the most common metabolic diseases, and its prevalence has been continuously increasing worldwide [1, 2, 3]. T2DM is characterized by hyperglycemia resulting from insulin resistance and deficiency in insulin secretion [4, 5]. Insulin resistance, which affects 65%–90% of obese people and 25%–45% of non‐obese people, is frequently caused by abnormalities in the insulin receptor or post‐receptor signaling, which impairs peripheral tissues' ability to absorb glucose [6]. Hyperglycemia makes T2DM patients susceptible to microvascular complications (nephropathy, retinopathy, and neuropathy) and macrovascular complications (cardiovascular and peripheral vascular disease) [7, 8, 9]. The prevalence of diabetes is increasing worldwide, primarily due to an increase in obesity, as systematic reviews and meta‐analyses have reported a high global prevalence of diabetic retinopathy and painful peripheral neuropathy associated with diabetes [10, 11, 12, 13].

Diabetic neuropathy (DN) is a clinical syndrome that affects the nervous system. This state results from sorbitol accumulation, enhanced aldose reductase activity, oxidative stress (OS), and disruptions in neural blood supply [14]. Another microvascular complication is diabetic retinopathy, which involves the destruction of retinal capillaries and intense retinal changes due to OS sensitivity [15]. It was previously believed to be only a microvascular complication of diabetes, but has now been identified as having a neurodegenerative component [16, 17, 18].

It has been proven that OS is a crucial factor in the onset of vascular complications in T2DM [19, 20]. Hyperglycemia elevates free fatty acid levels, increases the generation of reactive oxygen species (ROS), and reduces antioxidant activity, resulting in OS. Ultimately, the generated OS leads to endothelial dysfunction in the blood vessels of individuals with diabetes. The association between complications of diabetes and OS has been demonstrated by several reports, including the assessment of biomarkers indicating DNA damage and the presence of lipid peroxidation products such as MDA [21]. OS refers to the imbalance between the production of ROS and their neutralization by protective antioxidants. OS is a significant contributing factor in various disorders, including diabetes, cancer, viral infections, aging, neurodegenerative diseases, and cardiovascular complications [22, 23, 24, 25].

Vitamin D is involved in insulin secretion, calcium homeostasis, and the growth and differentiation of different cell types. In addition, it has antioxidant activity that can regulate the production of free radicals and enhance the glutathione peroxidase (GPx) and superoxide dismutase (SOD) levels in OS [26]. These mechanisms are regulated through specific vitamin D receptor (VDR). Also, the protective role of vitamin D against cancer and reducing its progression through limiting angiogenesis and metastasis, increasing apoptosis, controlling cell proliferation, modifying the immune system, and lowering OS has been suggested [27].

Therefore, any modifications to the VDR gene may have a notable impact on insulin levels, potentially affecting the pathogenesis of diabetes [28]. Binding 1,25 (OH)2‐D3 to its receptor, VDR, results in producing a transcriptional complex that regulates the associated vitamin D function gene transcriptions [29]. The VDR controls the influence of the 1,25 (OH)2‐D3 on the body through binding to a VDR heterodimer complex that activates the signal transduction [6, 30].

The VDR responsiveness can be impacted by genetic variants, including the *FokI* single‐nucleotide polymorphism [31, 32, 33]. The *FokI* T>C (rs2228570) polymorphism in exon 2 of the VDR gene results in the substitution of C for T, converting the ATG codon to ACG [34, 35]. The presence of the C allele (designated F) results in translation initiation at the second ATG site and the absence of the three amino‐terminal amino acids of the full‐length VDR protein (424 amino acids). This alteration affects the structure and function of the VDR protein. VDR is essential for insulin signaling pathways, and shorter VDR protein exhibits different transcriptional activity and binding affinity for vitamin D; this may lead to insulin resistance, a significant factor in T2DM [36, 37].

Some studies have indicated a stronger association in Asian populations, with the frequency of the ff genotype reported to be 4% among African Americans and 13% to 18% among Asians and Caucasians [37, 38, 39]. Individuals carrying the FF or Ff genotypes are more sensitive to the effects of vitamin D compared to those having the ff genotype [33]. The influence of VDR *FokI* polymorphism on insulin secretion and resistance and the serum level of HDL‐cholesterol has been reported [40]. The association of *FokI* polymorphism with microvascular complications of diabetes and its influence on dyslipidemia, OS, and body mass index (BMI) has not been reported among Iranians.

The increasing prevalence of diabetes and its serious microvascular complications, particularly neuropathy and retinopathy, highlight the need to elucidate their underlying genetic predispositions. While the critical role of vitamin D and its receptor (VDR) gene variants in cardiometabolic diseases, including diabetes, is increasingly recognized, a significant knowledge gap remains concerning the intricate association between VDR genetic variations, specifically the *FokI* polymorphism, and susceptibility to T2DM and its associated complications within the Iranian population. Moreover, the interplay of this polymorphism with dyslipidemia, OS, and BMI in this specific ethnic group remains unexamined. Therefore, the primary objective of this study was to investigate the association of the *FokI* gene variant of the VDR gene with T2DM, DN, and retinopathy in an Iranian cohort, and to determine its relationship with dyslipidemia, OS markers, and BMI. To comprehensively assess OS, serum levels of malondialdehyde (MDA), total antioxidant capacity (TAC), total oxidative status (TOS), reduced glutathione (GSH), GPx activity, and the oxidative stress index (OSI) value were evaluated.

## 2 Methods

The present study comprised 100 healthy individuals and 300 individuals with diabetes classified into subgroups: 100 T2DM without complication, 100 T2DM with DN, and 100 T2DM with diabetic retinopathy. The mean age of all patients, diabetes without complication, DN, and diabetic retinopathy, and controls was 56.8 ± 5.7 and 55.9 ± 6.0, 55.9 ± 5.8, 58.5 ± 4.8, and 52.8 ± 7.3 years (*p* < 0.001). The age range of patients was 45–67 years, and the age range of controls was 45–65 years. The male‐to‐female ratio among patients was 80:220, and in controls, it was 48/52. The duration of diabetes was 10.7 ± 4.3 years in diabetic patients without complication, 10.6 ± 4.4 years in DN patients, and 15.5 ± 5.4 years in patients with diabetic retinopathy. BMI was calculated as weight in kilograms divided by height in meters squared (kg/m^2^). The BMI was classified as BMI < 18.5 (underweight), BMI 18.5 to < 25 as normal range BMI, overweight (pre‐obese) with BMI 25–30, and obese with BMI > 30 kg/m^2^ [41].

The subjects were selected from the Diabetes Research Center at Taleghani Hospital of Kermanshah University of Medical Sciences, Kermanshah, Iran. The individuals with diabetes had a minimum 5‐year history of diabetes, and they did not exhibit any other systemic diseases [42]. The study was approved by the Ethics Committee of Kermanshah University of Medical Sciences (Approval ID: IR.KUMS.REC.1399.931), and written informed consent was provided by each individual.

The genomic DNA was extracted from white blood cells using the phenol‐chloroform method [43]. The purity and quantity of extracted DNA were detected by using the NanoDrop 2000 spectrophotometer (Thermo Fisher Scientific, Waltham, Massachusetts, USA).

The polymerase chain reaction (PCR) amplification of *FokI* T>C (rs2228570) was performed using the following forward primer of 5′‐AGC TGG CCC TGG CAC TGA CTC TGC TCT‐3′ and the reverse primer of 5′‐ATG GAA ACA CCT TGC TTC TTC TCC CTC‐3′ [35]. The amplification program was carried out using a thermocycler for 35 cycles at 94°C for 45 s, 66°C for 30 s, and 72°C for 45 s. Electrophoresis was performed on a 1.5% agarose gel with a minimum voltage of 120 V for at least half an hour to confirm the accuracy of gene amplification. The restriction fragment length polymorphism (RFLP) analysis was conducted after confirming successful amplification of the desired gene (validated by electrophoresis). Different genotypes were determined using the method of limited‐effect enzyme digestion. For the identification of the polymorphism *FokI* rs2228570, 5 units *FokI* restriction enzyme from Thermo Fisher (FastDigest FokI, Thermo Fisher Scientific, Cat# FD2144) was utilized. This enzyme recognizes the GGATG sequence and hydrolyzes it into 169 and 96 bp fragments.

### 2.1 Biochemical Analysis

The assessment of TAC in serum was performed using the ferric reducing ability of plasma (FRAP) method. In this method, the plasma's ability to reduce ferric ions (Fe^3+^‐TPTZ) to ferrous ions (Fe^2+^) and the subsequent production of a blue color at 593 nm (nm) was measured [42].

Using a colorimetric method, the TOS level was measured through ferric‐xylenol orange assay. In this process, oxidants present in the sample, such as hydrogen peroxide, convert ferrous ions to ferric ions. Ferric ions generate an orange‐colored complex with xylenol in a diluted acidic environment, and the intensity of this color is measurable. The OSI value was calculated by dividing TOS by TAC [42].

MDA is the final product of lipid breakdown under oxidative conditions. The MDA measurement was conducted using the thiobarbituric acid test. Its absorbance is measurable at 532 nm. The combination of GSH with fluorophores such as O‐Phthalaldehyde at pH 8.0 was employed for assessing the GSH level. GSH reacts specifically with OPA, generating a highly fluorescent product detectable at 350 nm, with emission peaking at 420 nm [42].

The assessment of GPx activity in the whole blood was conducted using the Randox kit (Randox Laboratories, Cat# RS505); the results were reported in units per milligram of hemoglobin (U/mgHb).

### 2.2 Statistical Analysis

Following data collection, statistical software SPSS 16 was utilized for the examination, analysis, and extraction of information. The frequency of the *FokI* genotypes among the study groups was compared using the *χ* ^2^ test and the Pearson's coefficient. To estimate an association between the *FokI* gene variants and the risk of diabetes and its microvascular complications, the odds ratios (ORs) and 95% confidence intervals (95% CIs) were calculated using SPSS logistic regression software. For evaluating the relationship between quantitative parameters and the genotypes within the study groups, the two‐tailed Student's *t*‐test and the analysis of variance (ANOVA) were employed, considering a significance level of *p* value < 0.05 to denote a statistically significant difference.

## 3 Results

Biochemical parameters in both genders of individuals with diabetes and controls are demonstrated in Table 1. Significantly higher levels of BMI, MDA, and lower levels of GSH, GPx, TAC, and TOS were observed in all individuals with diabetes than in controls. Comparing both genders in each group indicated significantly higher levels of BMI, OSI, and significantly lower levels of TAC in females than in males (Table 1). Also, comparing genders, we found significantly higher levels of BMI and OSI (TOS/TAC) and lower levels of TAC in women than men among both patients and controls (Table 1).

**TABLE 1:** Biochemical parameters in both genders of patients and controls.

Parameters	All diabetic patients	Patients	All controls	Controls
	*n* = 300	Females/(*n* = 220)	*n* = 100	Females/(*n* = 52)
		Males (*n* = 80), *p*		Males (*n* = 48), *p*
Age (years) Range	56.77 ± 5.66, < 0.001* 45–67	56.35 ± 5.64 45–67 57.91 ± 5.59, 0.035** 45–66	52.83 ± 7.33 45–65	50.92 ± 6.61 45–65 54.90 ± 7.58, 0.007** 45–65
BMI (kg/m^2^)	29.04 ± 4.27, < 0.001*	29.76 ± 4.41 27.04 ± 3.1, < 0.001**	26.93 ± 4.18	27.73 ± 4.18 26.07 ± 4.05, 0.047**
GSH (nmol/mL)	9.87 ± 4.14, 0.001*	9.76 ± 4.22 10.16 ± 3.94, 0.44	11.32 ± 3.36	11.10 ± 3.2 11.56 ± 3.54, 0.50
GPx (U/mgHb)	46.35 ± 10.49, < 0.001*	45.67 ± 9.95 48.25 ± 11.73, 0.082	59.91 ± 10.87	61.11 ± 11.44 58.61 ± 10.19, 0.25
TAC (nmol/mL)	742.75 ± 131.15, 0.046*	730.04 ± 128.80 777.69 ± 131.99, 0.006**	770.23 ± 113.67	739.64 ± 101.64 803.38 ± 117.68, 0.005**
TOS (nmol/mL)	3.80 ± 2.03, 0.023**	3.91 ± 2.18 3.51 ± 1.51, 0.079	4.18 ± 1.18	4.26 ± 1.24 4.09 ± 1.12, 0.47
MDA (nmol/mL)	6.82 ± 2.09, < 0.001*	6.92 ± 2.10 6.54 ± 2.05, 0.15	5.07 ± 1.62	5.01 ± 1.66 5.1 ± 1.58, 0.72
OSI (TOS/TAC)	0.0052 ± 0.0028, 0.24	0.0054 ± 0.0029 0.0046 ± 0.0022, 0.014**	0.0055 ± 0.0016	0.0058 ± 0.0017 0.0051 ± 0.0014, 0.039**

Abbreviations: GPx, glutathione peroxidase; GSH, glutathione; MDA, malondialdehyde; OSI, oxidative stress index; TAC, total antioxidant capacity; TOS, total oxidative status.

* Significantly different compared to controls.

** Significantly different compared to females.

The frequency of genotypes and alleles of *FokI* in all individuals with diabetes and healthy individuals is depicted in Table 2. The higher frequencies of the CC genotype (58.3%) and the C allele (78.2%) in all patients than controls (48, and 69%, respectively) were associated with 2.46 times (95% CI 1.45–4.19, *p* = 0.001) and 1.6‐fold (95% CI 1.12–2.98, *p* = 0.009) increased risk of T2DM, respectively. Also, the presence of the combined genotype of TC + CC compared to the TT genotype enhanced the risk of T2DM by 5.44‐fold (OR = 5.44, 95% CI 1.92–15.39, *p* = 0.001) (Table 2).

**TABLE 2:** Distribution of *FokI* genotypes and alleles in all diabetic patients and healthy individuals.

*FokI* genotypes and alleles	All diabetic patients	Healthy individuals	*χ* ^2^, *p*	OR (95% CI), *p*
	*n* = 300	*n* = 100		
	*n* (%)	*n* (%)		
Genotypes				
TT	6 (2)	10 (10)		
TC	119 (39.7)	42 (42)	9.3, 0.002	4.72 (1.61–13.78), 0.005
CC	175 (58.3)	48 (48)	13.63, < 0.001	2.46 (1.45–4.19), 0.001
TC + CC	294 (98)	90 (90)	12.5, < 0.001	5.44 (1.92–15.39), 0.001
Alleles				
T	131 (21.8)	62 (31)		
C	469 (78.2)	138 (69)	6.88, 0.009	1.6 (1.12–2.98), 0.009

*Note:* Comparison have been made with TT genotype and T allele.

The frequency of the *FokI* CC, the TC + CC genotypes, and the C allele were 56%, 99%, and 77.5% in individuals with diabetic neuropathy with neuropathy, which increased the risk of DN by 3.41‐, 11‐, and 3.68‐fold, respectively. Further, the *FokI* CC, the TC + CC genotypes, and the C allele frequencies in individuals with diabetic neuropathy with retinopathy were 58%, 98%, and 78%, which were associated with the elevated risk of diabetic retinopathy by 2.45, 5.44, and 1.59 times, respectively (Table 3).

**TABLE 3:** Distribution of *FokI* genotypes and alleles among diabetic patients with complications of neuropathy and retinopathy and without complication and in healthy individuals.

*FokI* genotypes and alleles	Diabetic patients without complication	Diabetic patients with neuropathy	Diabetic patients with retinopathy	Healthy individuals
	*n* = 100	*n* = 100	*n* = 100	*n* = 100
	*n* (%)	*n* (%)	*n* (%)	*n* (%)
Genotypes				
TT	3 (3)	1 (1)	2 (2)	10 (10)
TC	36 (36)	43 (43)	40 (40)	42 (42)
	*χ* ^2^ = 2.42, *p* = 0.12	*χ* ^2^ = 6.75, *p* = 0.009	*χ* ^2^ = 64.36, *p* = 0.037	
		OR = 10.23 (95% CI 1.25–83.53)	OR = 4.76 (95% CI 0.98–23.08)	
CC	61 (61)	56 (56)	58 (58)	48 (48)
	*χ* ^2^ = 5.03, *p* = 0.025	*χ* ^2^ = 7.97, *p* = 0.005	*χ* ^2^ = 6.24, *p* = 0.012	
	OR = 2.06 (95% CI 1.05–4.03)	OR = 3.41 (95% CI 1.2–9.71)	OR = 2.45 (95% CI 1.12–5.37)	
TC + CC	97 (97)	99 (99)	98 (98)	
	*χ* ^2^ = 4.03, *p* = 0.045	*χ* ^2^ = 7.79, *p* = 0.005	*χ* ^2^ = 5.67, *p* = 0.017	
	OR = 3.59 (95% CI 0.95–13.47)	OR = 11 (95% CI 1.38–87.64)	OR = 5.44 (95% CI 1.16–25.52)	90 (90)
Alleles				
T	42 (21)	45 (22.5)	44 (22)	62 (31)
C	158 (79)	155 (77.5)	156 (78)	138 (69)
	*χ* ^2^ = 5.19, *p* = 0.023	*χ* ^2^ = 3.68, *p* = 0.055	*χ* ^2^ = 4.15, *p* = 0.041	
	OR = 1.69 (95% CI 1.07–2.66)		OR = 1.59 (95% CI 1.01–2.49)	

*Note:* Comparison have been made with TT genotype and T allele between patients and controls.

The levels of BMI, OS parameters, and lipid profile have been compared between various genotypes of the VDR *FokI* in all individuals with diabetes (Table 4). Among patients, a significantly higher level of BMI was observed in the carriers of the TC + CC genotype (29.11 ± 4.28 kg/m^2^) than in the TT genotype (25.87 ± 1.99 kg/m^2^, *p* = 0.009). Also, among these patients, carriers of the combined genotype of TC + CC had significantly higher levels of TOS (3.83 ± 2.04 nmol/mL, *p* < 0.001) and triglycerides (TG, 145.55 ± 67.96 mg/dL, *p* = 0.048) than the TT genotype (2.63 ± 0.45, 108.83 ± 34.70, respectively) (Table 4).

**TABLE 4:** Comparison of BMI, oxidative stress parameters, and lipid profile in diabetic patients with various *FokI* genotypes.

Parameters	TT (*n* = 6)	TC+CC (*n* = 294)	*p*
BMI (kg/m^2^)	25.87 ± 1.99	29.11 ± 4.28	0.009
Glutathione (nmol/mL)	9.26 ± 4.71	9.89 ± 4.14	0.763
GPx (U/mgHb)	46.05 ± 13.12	46.37 ± 10.47	0.956
TAC (nmol/mL)	692.22 ± 123.67	743.78 ± 131.29	0.357
TOS (nmol/mL)	2.63 ± 0.45	3.83 ± 2.04	< 0.001
MDA (nmol/mL)	6.80 ± 1.01	6.83 ± 2.10	0.969
OSI (TOS/TAC)	0.0039 ± 0.0013	0.0052 ± 0.0028	0.059
Cholesterol (mg/dL)	175.67 ± 45.59	158.46 ± 31.77	0.39
Triglycerides (mg/dL)	108.83 ± 34.70	145.55 ± 67.96	0.048
HDL‐C (mg/dL)	43.67 ± 6.50	40.14 ± 9.30	0.24
LDL‐C (mg/dL)	99.50 ± 32.40	85.63 ± 24.02	0.34

Abbreviations: BMI, body mass index; GPx, glutathione peroxidase; MDA, malondialdehyde; OSI, oxidative stress; TAC, total antioxidant capacity; TOS, total oxidative status index.

Among all studied individuals in the presence of the combined genotype of the *FokI* TC + CC compared to the TT genotype, there was a higher level of TOS (3.92 ± 1.88 nmol/mL) in the carriers of TC + CC (*n* = 384) than in TT carriers (*n* = 16, 3.32 ± 0.87 nmol/mL, *p* = 0.02).

Comparing biochemical parameters in various *FokI* genotypes based on gender, it was observed that among women with the FokI CC genotype (*n* = 150, 48.07 ± 11.58 U/mgHb), there was a significantly lower activity of GPx compared to those with the TT genotype (*n* = 8, 58.6 ± 11.75 U/mgHb, *p* = 0.039).

## 4 Discussion

The OS serves as a key element in comprehending the intricate mechanisms leading to the onset and progression of diabetes and its associated complications [44]. Studies have shown that there are positive relationships between OS and diabetes and its complications [24]. Among these contributing factors, MDA emerges as a byproduct of lipid oxidation. A study conducted by Smiriti et al. investigated the role of salivary MDA in assessing OS among individuals with diabetes. Their study demonstrated a significant discrepancy in MDA concentrations between serum and saliva of individuals with diabetes compared to the control group, indicating elevated MDA levels among individuals with diabetes [45]. Our findings revealed a significant discrepancy in MDA levels between individuals with diabetes and the control group, with markedly elevated MDA concentrations observed in the patient population compared to the control subjects.

In the present study, the comparison of GSH levels between all individuals with diabetes and the control group demonstrated a significantly lower GSH level in all individuals with diabetes compared to the control group. Similarly, the assessment of GPx levels between all individuals with diabetes and the control group revealed a significantly lower GPx level in all individuals with diabetes compared to the control group. In a study, antioxidant enzymes were measured in T2DM patients and healthy individuals, and a considerable reduction in GPx and GSH levels was observed in individuals with diabetes compared to the controls. This study suggested that in hyperglycemia, excess glucose prefers to enter the polyol pathway, consuming NADPH required for GSH regeneration by glutathione reductase. Consequently, the depletion of GSH and reduced GPx activity might be attributed to the reduction of GSH in individuals with diabetes, as GSH serves as the substrate for GPx [46]. In another study among newly diagnosed individuals with diabetes, it was found that the GSH levels in newly diagnosed individuals with diabetes were reduced compared to controls, indicating the entrapment of free radicals. Moreover, GPx levels in these patients were also diminished due to the reduction in GSH levels. This association of reduced GPx and GSH levels in individuals with diabetes aligns with the outcomes observed in our study [47].

In our study, the examination of TAC between individuals with diabetes and the control group revealed a significant reduction in the TAC levels in individuals with diabetes compared to the controls. In a study by Etienne and colleagues in Brazil, the TAC levels were diminished in diabetic and DN patients compared to the controls [48]. Moreover, the examination of TOS between all individuals with diabetes and the control group indicated a significant decrease in TOS levels in all individuals with diabetes compared to the controls. However, higher TOS levels in individuals with diabetes with cataracts compared to non‐diabetic cataract patients have been reported [49]. This finding is in contrast to some previous studies that have reported elevated TOS in diabetic populations [50]. Diabetes presents a complex relationship between TOS and TAC. Although OS is typically high, the actual levels of TOS and TAC may differ based on the presence and severity of disorders, such as diabetic nephropathy [50, 51]. However, the observed difference could be due to TOS assay methods that exhibit different sensitivities to specific oxidative species, potentially leading to an underestimation of total OS in the diabetic group. In addition, a subset of diabetic participants may have received antioxidant therapy, including vitamin E and alpha‐lipoic acid, which can significantly reduce TOS levels in diabetic patients. Finally, the specific characteristics of our sample population, such as disease duration, glycemic control (HbA1c level), age, and other comorbidities, may be significantly different from previous studies.

The T (f) allele of the FokI polymorphism (T>C) encodes a longer and less transcriptionally active VDR protein, while the C (F) allele results in a shorter, more active form [35]. The variants of the VDR gene affect the binding of vitamin D to its receptor, and vitamin D function that could result in diseases such as asthma and diabetes mellitus [52].

In a large prospective study that included 14,709 patients with T2DM, higher serum levels of 25(OH)‐D were significantly associated with a lower risk of diabetic retinopathy, DN, and diabetic nephropathy. Some mechanisms have been suggested for the protective role of vitamin D on the occurrence of microvascular complications of diabetes, such as the prevention of neovascularization, inhibition of renin‐angiotensinangiotensin‐aldosterone system, and prevention of diabetic nerve damage by adequate levels of vitamin D. Also, vitamin D could maintain vascular homeostasis, improve glucose and lipid metabolism, and increase antioxidant defense and immune system function. However, no modifying effect of VDR genetic variants was observed [53].

*The protective role of VDR* in glucose metabolism reprogramming has been suggested through the phosphorylation of pyruvate dehydrogenase E1 subunit alpha 1 inhibition. This subunit is the key regulatory site of the pyruvate dehydrogenase complex, catalyzing the conversion of pyruvate into acetyl‐CoA within mitochondria [54]. Consequently, vitamin D deficiency and dysfunction of its receptor, VDR, can lead to decreased glucose tolerance and impaired insulin secretion, ultimately resulting in insulin resistance. VDR polymorphisms, which mediate the genomic effects of vitamin D, may contribute to the pathogenesis of T2DM and its complications by influencing the secretory capacity of pancreatic beta cells and vitamin D concentrations. Furthermore, vitamin D is known to initiate the transcription of the human insulin gene, and vitamin D insufficiency may be associated with reduced insulin secretion in T2DM [55].

Variations in the gene sequences, including single‐nucleotide polymorphisms, may lead to individual differences in traits such as susceptibility to disease, as a molecular marker for multiple myeloma risk [56] or response to diabetes treatment [57].

The present study indicated that CC genotype and the C allele of *FokI* were associated with 2.46 times and 1.6‐fold increased risk of T2DM, respectively. Also, the higher frequency of the *FokI* CC and the TC + CC compared to the TT genotype and the C allele in individuals with diabetes with neuropathy compared to healthy individuals enhanced the risk of DN by 3.41‐, 11‐, and 3.68‐fold, respectively. Further, the presence of the *FokI* CC, the TC + CC genotypes, and the C allele elevated the risk of diabetic retinopathy by 2.45, 5.44, and 1.59 times, respectively.

Several reports discuss the impact of FokI polymorphism on the risk of developing diabetes. Taneja et al. conducted a study involving 100 T2DM patients and 100 healthy individuals. They found that the genotype distributions differed significantly between patients and controls. They also noted a significantly higher frequency of the CC genotype among individuals with diabetes and an increased risk of diabetes [58]. In another study conducted by Mohamed et al. in Egypt involving 200 postmenopausal women, the researchers observed a significant increase in the frequency of the FF genotype among individuals with diabetes compared to the control group. Among the diabetic group, the allele F frequency increased compared to the non‐F allele [59]. Also, Alfaqih et al. [60] showed a higher frequency of the C allele in T2DM patients than in controls and suggested that the C allele of the *FokI* rs2228570 may be a high‐risk allele for T2DM among the Jordanian population. However, no association was found between the VDR *FokI* polymorphism and the risk of T2DM among postmenopausal Brazilian women [34]. Also, in Malecki et al.'s study involving 308 T2DM patients and 240 controls in the Polish population, no association was found between the presence of the *FokI* polymorphic genotype and an increased risk of T2DM [61]. Further, among the Slovak population with T2DM, the VDR *FokI* was not associated with the risk of diabetes [57].

In a meta‐analysis that consisted of eight thousand eleven diabetic patients and 1635 normal controls, an association between VDR gene variants, including VDR *FokI*, with diabetic nephropathy and diabetic retinopathy susceptibility was reported [62].

In the present study, individuals with diabetes with a combined genotype of TC + CC had significantly higher levels of TG than the TT genotype. Also, in the presence of the combined genotype of TC + CC compared to the TT genotype, the level of TOS significantly increased. It seems the VDR *FokI* variants influence dyslipidemia and OS, which are both involved in the risk of diabetes mellitus development. In diabetes mellitus patients, there is dyslipidemia with abnormal lipid profiles characterized by increased levels of TG and LDL‐C, along with low levels of HDL‐C [52]. In Jordanian Arabic origin with T2DM patients, the *VDR ApaI* and *BsmI* genotypes were associated with HDL‐C levels and retinopathy [52].

In the present study, we demonstrated significantly higher BMI levels in individuals with diabetes than in controls; we also indicated that the *FokI* TC + CC genotype, compared to the TT genotype, results in higher BMI levels in individuals with diabetes. Further, among women in the presence of the *FokI* CC genotype, there was a lower activity of GPx than in the TT genotype. Association between obesity and *FokI* polymorphism has been suggested [57]. It was suggested that obesity is a chronic low‐grade inflammation status that, through elevation of OS, is involved in the progression of diabetes [63].

The present study found significantly higher levels of BMI and OSI as well as significantly lower levels of TAC in females compared to males among both individuals with diabetes and controls. These findings suggest that the risk factors of developing T2DM, specifically obesity and OS, are more prevalent in women than in men. Additionally, obesity is associated with increased OS in diabetes mellitus. The absence of estrogen in women due to menopause contributes to the development of T2DM. In women with diabetes, there is a stronger association between obesity and diabetes than in men, likely due to the reduction of estrogen and the increasing insulin resistance associated with aging and menopause [64]. Obesity, characterized by low‐grade chronic systemic inflammation in adipose tissue, increases the levels of pro‐inflammatory markers and OS in affected individuals. In obese individuals, the depletion of both enzymatic and non‐enzymatic antioxidant sources heightens susceptibility to oxidative damage [65]. Excess adipose tissue acts as a source of pro‐inflammatory cytokines, enhancing the production of reactive oxygen and nitrogen species and increasing the rate of lipid peroxidation, which in turn leads to OS. The levels and the activity of components of the antioxidant defense system are diminished in obesity, further increasing the risk of oxidative tissue damage [66].

It has been suggested that T2DM‐associated damage may be different according to the age and gender of the diabetic patients. Many women, in association with hormonal changes, decrease in estrogen levels, and being overweight or obese after 45 years of age. In obese subjects in association with insulin resistance, the biomarkers of OS appear. Obesity is related to the higher production of H_2_O_2_ by mitochondria and a lower ratio of GSH/oxidized glutathione disulfide in skeletal muscle that has a negative correlation with BMI [67].

## 5 Conclusion

This study indicates that in individuals with diabetes, there are higher OS levels reflected in higher levels of BMI, MDA, and lower levels of GPx, GSH, and TAC compared to healthy individuals. Also, we found that the *FokI* CC genotype and the *FokI* C allele are associated with an increased risk of T2DM and its complications. The combined TC + CC genotype was associated with significantly higher levels of TG, TOS, and BMI compared to the TT genotype in individuals with diabetes, highlighting the influence of VDR *FokI* variants on dyslipidemia, OS, and BMI—all established risk factors for diabetes development. We found that the VDR *FokI* variants influenced dyslipidemia, OS, and BMI, the risk factors of diabetes mellitus development. Furthermore, the present study detected significantly higher levels of BMI and OSI and a substantially lower level of TAC in females than in males among both individuals with diabetes and controls. It seems the risk factors for developing T2DM, obesity, and OS among women are more prevalent than in men. Although these findings highlight the role of VDR and OS variants in the pathogenesis of T2DM and its complications, further studies with larger and more diverse populations are needed to validate these associations and to investigate the underlying mechanisms through which VDR FokI variants influence metabolic parameters and OS. Investigating the interaction between genetic predisposition, lifestyle factors, and environmental influences could provide a more comprehensive understanding of T2DM risk. Future research could also explore the potential of targeted interventions, such as vitamin D supplementation or antioxidant therapies, to reduce OS and decrease the risk of T2DM and its complications, particularly in vulnerable populations. These future directions hold promise for translating these findings into effective strategies for managing and reducing the burden of T2DM on the healthcare system.

## 6 Limitations of the Study

The limitations of the present study were the absence of information related to the serum vitamin D level and lifestyle parameters in the studied individuals.

## Author Contributions

Armin Sharifi and Mahsa Nouri performed experiments and wrote the original draft of the manuscript. Ebrahimi Shakiba and Zahra Ghorbani performed data analysis. Zohreh Rahimi performed data analysis and interpreted the results, and critically revised the manuscript.

## Ethics Statement

The study was approved by the Ethics Committee of Kermanshah University of Medical Sciences (Approval ID: IR.KUMS.REC.1399.931), and written informed consent was provided by each individual.

## Conflicts of Interest

The authors declare no conflicts of interest.

## Data Availability

All data generated or analyzed during this study are included in this article. Further enquiries can be directed to the corresponding author.
